# Photoconductive terahertz generation from textured semiconductor materials

**DOI:** 10.1038/srep23185

**Published:** 2016-03-16

**Authors:** Christopher M. Collier, Trevor J. Stirling, Ilija R. Hristovski, Jeffrey D. A. Krupa, Jonathan F. Holzman

**Affiliations:** 1 Integrated Optics Laboratory, The University of British Columbia, Kelowna, BC, V1V 1V7, Canada

## Abstract

Photoconductive (PC) terahertz (THz) emitters are often limited by ohmic loss and Joule heating—as these effects can lead to thermal runaway and premature device breakdown. To address this, the proposed work introduces PC THz emitters based on textured InP materials. The enhanced surface recombination and decreased charge-carrier lifetimes of the textured InP materials reduce residual photocurrents, following the picosecond THz waveform generation, and this diminishes Joule heating in the emitters. A non-textured InP material is used as a baseline for studies of fine- and coarse-textured InP materials. Ultrafast pump-probe and THz setups are used to measure the charge-carrier lifetimes and THz response/photocurrent consumption of the respective materials and emitters. It is found that similar temporal and spectral characteristics can be achieved with the THz emitters, but the level of photocurrent consumption (yielding Joule heating) is greatly reduced in the textured materials.

The advent of mode-locked ultrafast pulsed lasers enabled many scientific pursuits including ultrafast material characterization studies^1^,^2^, surgical instrumentation advancements^3^, and terahertz (THz = 10^12^ Hz) generation and detection^4^. Of particular interest is the THz spectrum, with frequencies spanning 0.1–10 THz (corresponding to wavelengths spanning 30–3,000 μm), which has received much attention over the past few decades^5^,^6^,^7^,^8^,^9^. Terahertz technologies have provided advancements for applied science applications, such as tomography^10^, semiconductor characterization^11^,^12^, security^13^, and biomedical applications, including medical imaging^14^, diagnostics^15^, and DNA analyses^16^. These applications have developed over the past years along with THz generation and detection advancements. The generation of THz is usually implemented either as continuous-waves, through difference-frequency generation within electro-optic crystals^17^ or through heterodyne mixing within semiconductors^18^, or as pulses, through optical rectification within electro-optic crystals^19^ or through excited charge-carriers within photoconductive (PC) THz emitters^4^.

Photoconductive THz emitters are of particular interest as the strength of the emitted THz electric field can be scaled to large levels with increasingly high bias voltage amplitudes and pump optical fluences^20^. Photoconductive THz emitters radiate a THz electric field pulse by way of accelerating charge-carriers within the first few picoseconds of photoexcitation. However, these charge-carriers often have lifetimes that are much longer than the THz pulse durations, creating residual photocurrents which lead to unnecessarily large ohmic loss and Joule heating. The Joule heating prevents the THz electric field from being scaled up by simply increasing the bias voltage amplitude or pump optical fluence^21^, as the onset of thermal runaway (for which the photocurrent grows nonlinearly with the bias voltage amplitude) is inversely proportional to the pump optical fluence^22^. Likewise, the average THz beam power is prevented from being scaled up by simply increasing the repetition rate of the incident pump pulses^23^, as Joule heating increases proportionally to the repetition rate of the incident pump pulses.

In response to the above challenges, researchers have investigated large-area PC THz emitters, to scale up the emitted THz electric field by increasing the optically-active area of the PC THz emitter^24^. However, these systems can suffer from considerable Joule heating. In addition, PC THz emitter areas must often be made small, for applications with spatial constraints such as lab-on-a-chip systems^25^. The small area of such PC THz emitters dictates that the Joule heating of the PC THz emitters must be minimized, as the threshold voltage for thermal runaway is inversely related to emitter area^22^.

The solutions proposed to the above problems can be complicated. The solutions include intricate electrode designs^26^,^27^, patterning of the active area^28^, heat sink integration^29^, low-temperature substrate growth^22^, water-cooling^30^, substrate irradiation^21^, laser-ablation^31^, use of porous substrates^32^, and photo-generated dipole enhancement^33^. With this in mind, this work investigates solutions to minimize the photocurrent consumption of PC THz emission. This requires the ability to understand (and ultimately exploit) charge-carrier dynamics within semiconductor materials. It is desired to have the charge-carrier lifetime be as short as possible, to reduce the steady-state photocurrent, and this is accomplished by photoinjected charge-carriers in semiconductor materials with high densities of surface states. There are two investigated approaches to accomplish this charge-carrier lifetime reduction. The first approach for reducing the charge-carrier lifetime involves the preferential photoinjection of charge-carriers into InP at the semiconductor surface through the use of high-energy pump photons. Although this first approach significantly shortens the charge-carrier lifetime, it is found that the high-energy pump photons lead to an elongated rise-time, which is poorly-suited to THz generation. The second approach for reducing the charge-carrier lifetime involves the use of textured semiconductor materials. This second approach is found to be effective. The texturing increases the optically-excited surface area of the PC THz emitters and decreases the charge-carrier lifetime through increased contributions from surface recombination^34^,^35^. This work investigates the ultrafast material response and the THz response of non-, fine-, and coarse-textured InP materials. The relative surface areas (quantifying roughness) are measured by way of scanning electron microscope (SEM) images, the charge-carrier lifetimes are measured by way of a pump-probe setup, and the THz responses (in terms of radiated THz electric field and photocurrent consumption) are measured by way of a THz setup. It is found that similar temporal and spectral characteristics can be achieved with the THz emitters, using the three differently textured InP materials, but the level of photocurrent consumption (yielding Joule heating) is greatly reduced in the materials with rougher textures. Comments and comparisons are made on the application of these findings to future implementations of PC THz emitters.

## Results

The overarching desire to minimize the photocurrent consumption during PC THz emission requires the ability to understand (and ultimately exploit) the charge-carrier dynamics within the underlying semiconductor. With this in mind, the radiated THz electric field, *E*_THz_(*t*), that is emitted from a PC THz emitter can be linked to the charge-carrier density, *n*(*t*), by noting the proportionality between *E*_THz_(*t*) and the derivative of the conductivity. This gives


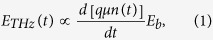


where *q* is the elementary charge constant, *μ* is the mobility, and *E*_b_ is the bias electric field. During photoexcitation by the pump beam, the charge-carrier density rises rapidly, and these charge-carriers become subject to a mobility that is ideally large and constant—as this condition leads to an equally rapid rise in the conductivity and broadband THz emission. In general, the photoexcitation wavelength and material must be selected together with careful consideration to energy relaxation and its manifestation as a transient mobility^36^. Following photoexcitation by the pump beam and emission of the THz electric field pulse, the conductivity typically remains high, over a duration defined by the charge-carrier lifetime of the material. This leads to a residual photocurrent that is undesirable—as it only contributes to Joule heating within the material. In general, one wishes to decrease the charge-carrier lifetime and lessen the Joule heating.

The Joule heating, *P*_H_, in a PC THz emitter can be quantified by integrating the electrical power over the charge-carrier lifetime, *τ*, and dividing the result by the period, *T*_0_, of the pump pulses. In carrying this out with *τ* ≪ *T*_0_, the Joule heating is found to be


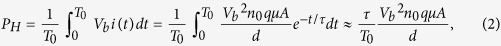


where *V*_b_ is the bias voltage amplitude, *n*_0_ is the initial charge-carrier density, *d* is the spacing between electrodes, and *A* is the cross-sectional area for transport of charge-carriers across the active region (being the product of the width of the electrodes and the depth of the charge-carriers). The linear proportionality that forms here between *P*_H_ and *τ* makes it apparent that a short charge-carrier lifetime is desirable to reduce the effects of Joule heating.

The stated goal for a reduction in the charge-carrier lifetime can be met by promoting surface recombination^34^,^35^. Surface recombination reduces the charge-carrier lifetime according to


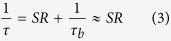


where *S* is the surface recombination velocity, *R* is the surface-area-to-volume ratio, and *τ*_b_ is the (exceedingly long) bulk charge-carrier lifetime of the material. For the proposed work, pertaining to InP material, it is desired to have the charge-carrier lifetime be as short as possible, to reduce the steady-state photocurrent, and this is accomplished by photoinjecting charge-carriers into regions of the material with high densities of surface states. There are two approaches considered in this work to accomplish this.

**Figure 1:**
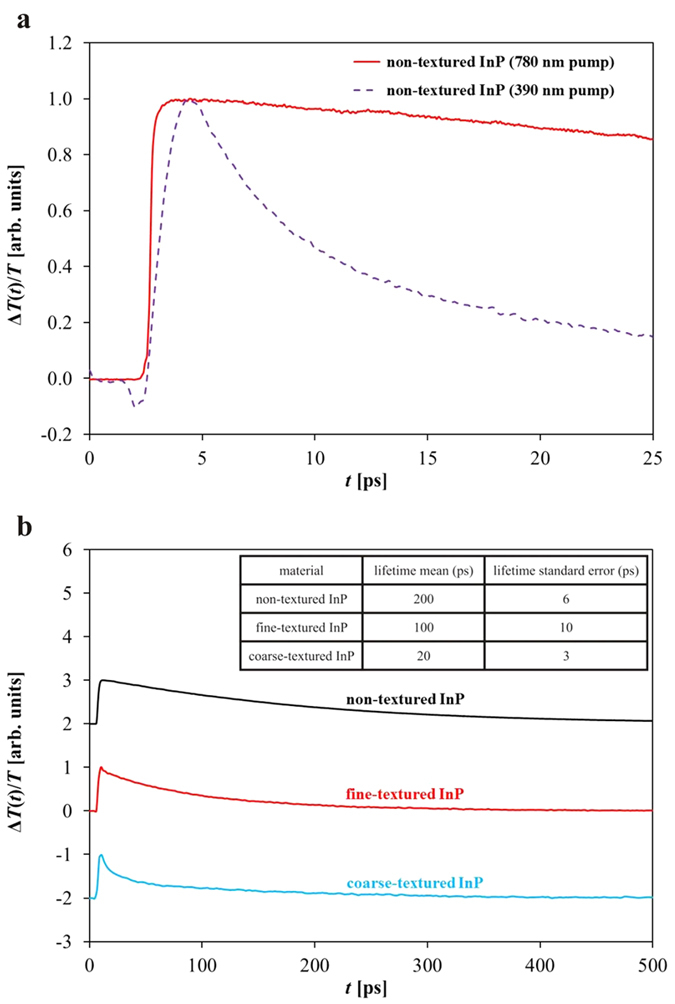
Differential transmission signals are shown for (**a**) non-textured InP material at 780 and 390 nm pump and 1550 nm probe wavelengths and for (**b**) non-, fine-, and coarse-textured InP materials at 780 nm pump and 1550 nm probe wavelengths. The (normalized) differential transmission pump-probe signals in (**a**) are plotted as a function of time and show an elongated rise-time for the 390 nm pump results compared to the 780 nm pump results. The (normalized) differential transmission pump-probe signals in (**b**) are plotted as a function of time and are curve-fit to decaying exponential functions to define the charge-carrier lifetime of each measurement. The results of (**b**) are shifted vertically for illustration purposes. The inset in (**b**) shows a table of the lifetime mean and lifetimes standard error for the non-, fine-, and coarse-textured InP materials for six measurement locations on each material.

The first approach for reducing the charge-carrier lifetime involves the preferential photoinjection of charge-carriers at the semiconductor surface through the use of high-energy pump photons. The energy of the pump photons is selected to have a penetration depth that is on the order of tens of nanometers, which leads to the preferential photoinjection of charge-carriers at the semiconductor surface. Thus, the charge-carriers become subject to an increased presence of surface states, yielding an increased surface-area-to-volume ratio and a decreased charge-carrier lifetime. With this in mind, pump-probe analyses are carried out to characterize the charge-carrier dynamics of InP with low-energy (780 nm) pump photons and high-energy (390 nm) pump photons. The results are shown in Fig. 1(a) as differential transmission curves, with a solid red line for the low-energy (780 nm) pump photons and a dashed violet line for the high-energy (390 nm) pump photons. The results do show a lower charge-carrier lifetime for the high-energy pump photons, compared to that of the low-energy pump photons, but there is also an increase in the rise-time of the photocurrent for the high-energy pump photons. The photocurrent is seen to rise over a timescale of 1 ps, rather than the duration of the incident optical pulse, and this delay is due to energy relaxation of the charge-carriers. The initially hot electrons must relax down to the bottom of the central *Γ* valley, via scattering with phonons, and it is only after this delay that they have a sufficiently high mobility to undergo transport in the external electric field. The authors selected semi-insulating- (SI-) InP over SI-GaAs for this investigation, because these InP and GaAs materials exhibited similar photocurrent rise-times under 780 nm pump photoexcitation, but the InP material exhibited faster surface recombination—with a surface recombination velocity of *S* = 10^6^ cm/s, in agreement with literature values^37^. Overall, it can be concluded that the use of high-energy pump photons for PC THz emitters is unacceptable given that the delay in the rise-time of the photocurrent greatly diminishes the THz spectral bandwidth—in addition to the fact that the excess energy of the charge-carriers introduces unnecessary Joule heating.

**Figure 2:**
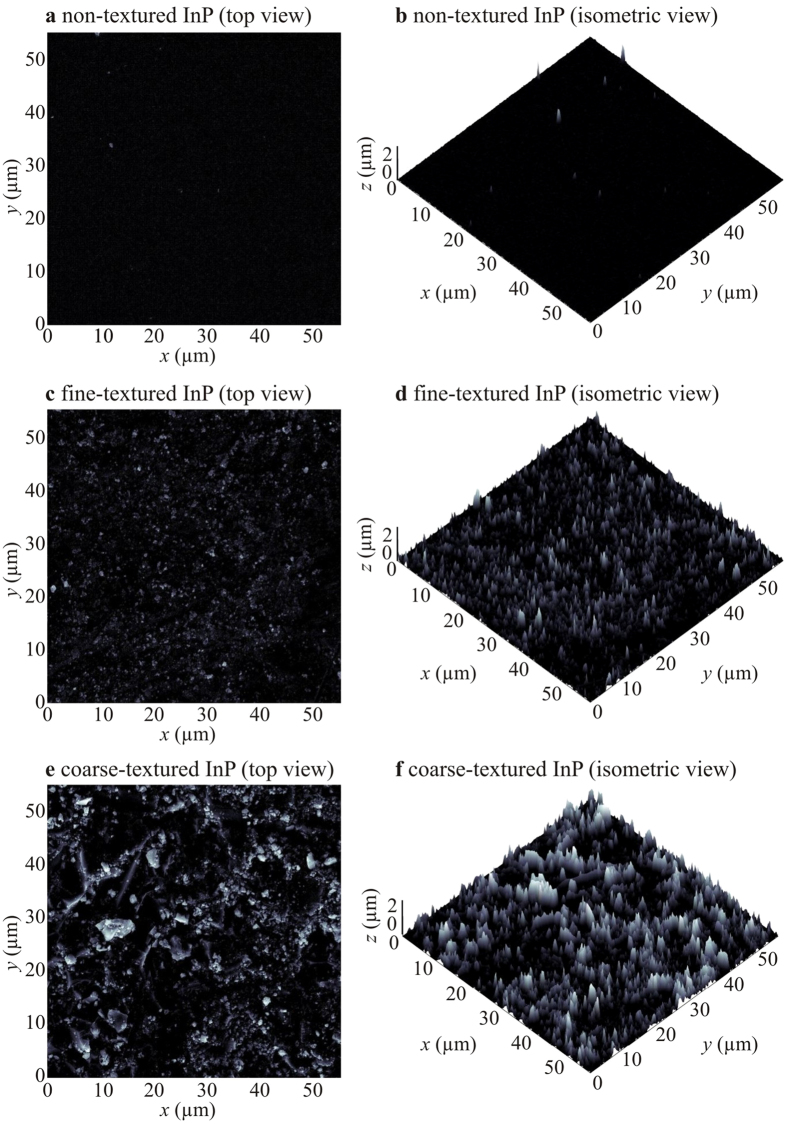
Top view SEM topographies for non-, fine-, and coarse-textured InP materials are shown in (**a**,**c**,**e**), respectively. Isometric view SEM topographies for respective non-, fine-, and coarse-textured InP materials are shown in (**b**,**d**,**f**), respectively. The relative surface areas of the non-, fine-, and coarse-textured InP materials are 1.0 ± 0.1, 2.9 ± 0.4, and 4.3 ± 0.6, respectively.

The second approach for reducing the charge-carrier lifetime uses textured semiconductor materials. Texturing of a planar semiconductor surface can increase the presence of surface states, which ultimately increases *R* according to equation (3) and decreases *τ*. The resulting reduction in the steady-state photocurrent leads to decreased Joule heating. This investigation uses non-, fine-, and coarse-textured InP materials to define three distinct regimes for the texturing—each with a characteristic length-scale of the texturing, *l*, that is compared to the *L* ≈ 2 μm diffusion length of the charge-carriers. The non-textured InP material has a negligible characteristic length-scale of the texturing, i.e., *l* ≈ 0 μm ≪ *L*. The fine-textured InP material has an intermediate characteristic length-scale of the texturing, i.e., *l* ≈ 1 μm < *L*. The coarse-textured InP material has a large characteristic length-scale of the texturing, i.e., *l* ≈ 2 μm ≈ *L*. To fabricate the non-, fine-, and coarse-textured InP materials, a polished SI-InP sample is used as the non-textured InP material, and varying grit-sizes of optical polishing films are used to transform the InP non-textured material into fine- and coarse-textured InP materials. The InP sample is mechanically polished with a 6 μm grit diamond polishing film (Thorlabs LF6D) and a 30 μm grit diamond polishing film (Thorlabs LF30D) to produce the fine-textured and coarse-textured InP materials, respectively. Relative surface areas are used to define the materials—as the unit-less ratio of surface areas between the material of interest and the non-textured InP material. The relative surface area for each material is found through analyses of SEM images. (Representative imaging sites are selected.) The SEM images are analysed with software that formulates three-dimensional greyscale topographies of the SEM images, and surface areas are extracted from these topographies. Representative images of non-, fine-, and coarse-textured InP materials are shown in Fig. 2. Top view SEM topographies are shown in Fig. 2(a,c,e), and isometric view SEM topographies are shown in Fig. 2(b,d,f) for representative SEM topographies of non-, fine-, and coarse-textured InP materials, respectively. The relative surface areas of the non-, fine-, and coarse-textured InP materials are found to be 1.0 ± 0.1, 2.9 ± 0.4, and 4.3 ± 0.6, respectively, based on ten sites per material.

The ultrafast material response of the non-, fine-, and coarse-textured InP materials are analysed with a pump-probe setup to determine the corresponding charge-carrier lifetimes. Representative (normalized) differential transmission, Δ*T*(*t*)/*T*, curves are shown as a function of time, *t*, in Fig. 1(b) for the non-, fine-, and coarse-textured InP materials as black, red, and blue solid lines, respectively. The results are shifted vertically for illustration purposes. The authors note that the differential transmission results shown in this figure exhibit positive polarity, and this positive polarity agrees with theoretical calculations for (Drude) free-carrier absorption and dispersion in which the decreasing refractive index of free-carrier dispersion dominates. (Further details are included in the Methods section.) The Δ*T*(*t*)/*T* curves are curve-fit with decaying exponential functions to define the charge-carrier lifetimes. The charge-carrier lifetimes are found to be 200 ± 6, 100 ± 10, and 20 ± 3 ps, for the non-, fine-, and coarse-textured InP materials, respectively, and are shown in the table in the Fig. 1(b) inset. The charge-carrier lifetimes are defined here as the charge-carrier lifetime mean plus or minus the charge-carrier lifetime standard error—based on six measurement sites per material. The charge-carrier lifetime of the non-textured InP is in approximate agreement with the literature value^38^.

**Figure 3:**
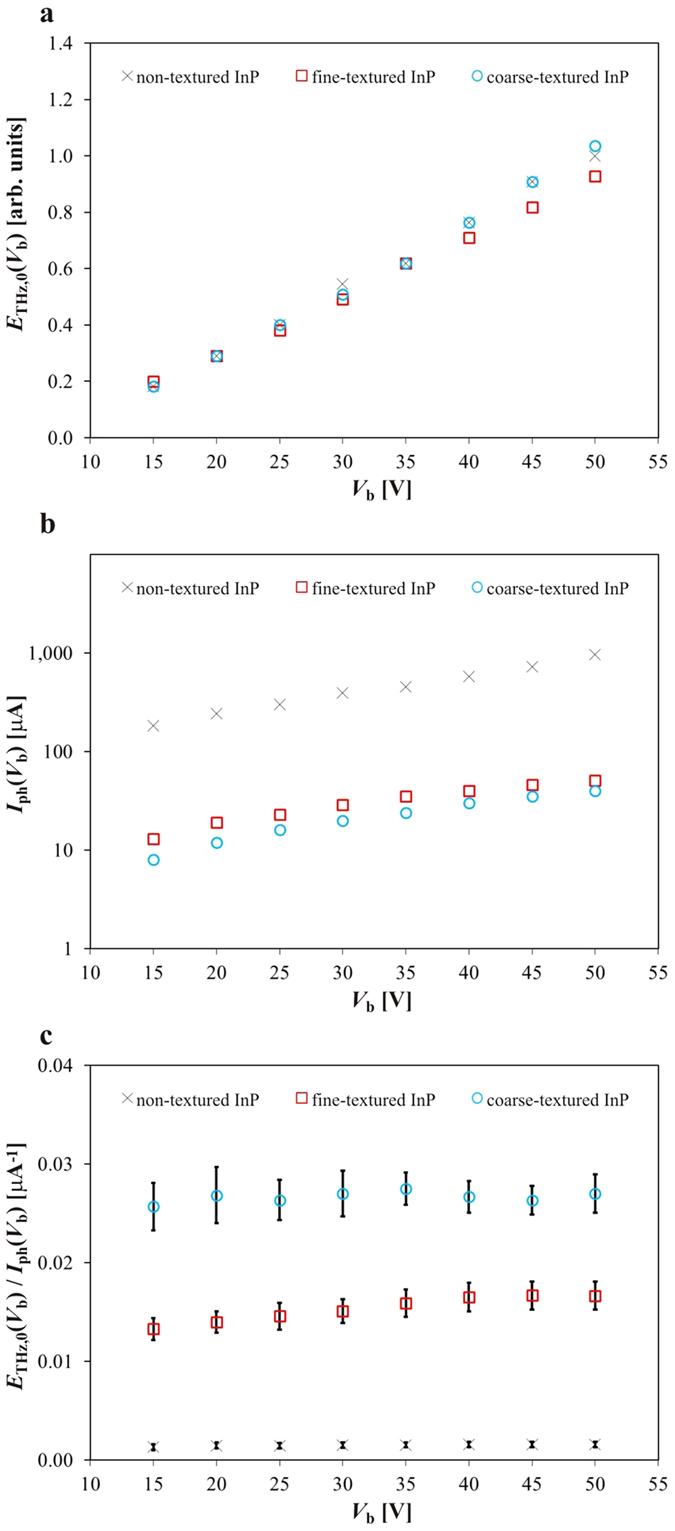
The THz responses of the non-, fine-, and coarse-textured InP PC THz emitters are shown. In (**a**) THz electric field measurements are taken as a function of applied voltage amplitude while each PC THz emitter is set to produce a similar THz electric field amplitude. The THz electric field amplitude values are normalized with respect to the non-textured InP emitter’s response at an applied voltage amplitude of 50 V to produce normalized THz electric field values. In (**b**) the corresponding photocurrent is measured as a function of applied voltage amplitude. In (**c**) the ratio of the normalized THz electric field amplitude over photocurrent is plotted as a function of bias voltage amplitude. The error bars represent the standard error of the ratio of normalized THz electric field amplitude over the photocurrent.

Now that the charge-carrier lifetime of the non-, fine-, and coarse-textured InP materials are measured, the THz response (being the radiated THz electric field and corresponding photocurrent consumption) of the non-, fine-, and coarse-textured InP PC THz emitters can be determined with a THz setup. Initially, measurements are taken with each PC THz emitter set to produce a similar THz electric field amplitude (normalized to the non-textured InP PC emitter’s THz electric field amplitude at a bias voltage amplitude of 50 V), with amplitude standard errors of 0.26, 0.04, and 0.38 for the non-, fine-, and coarse-textured InP PC THz emitters, respectively, based on six measurement sites per material. This is shown in Fig. 3(a) with normalized THz electric field amplitude, *E*_THz,0_(*V*_b_), plotted as a function of the bias voltage amplitude, *V*_b_, for non-, fine-, and coarse-textured InP PC THz emitters, shown respectively as black crosses, red squares, and blue circles. Each emitter has a consistent 100 μm electrode gap size. Simultaneously with the THz electric field measurements, the photocurrent, *I*_ph_(*V*_b_), is measured as a function of bias voltage amplitude, *V*_b_, and is plotted in Fig. 3(b) for non-, fine-, and coarse-textured InP PC THz emitters, shown respectively as black crosses, red squares, and blue circles. The non-textured InP PC THz emitter has a very large photocurrent while the InP fine-textured PC THz emitter has a marginal photocurrent, and the InP coarse-textured PC THz emitter has a very small photocurrent. The dark resistance of the non-, fine-, and coarse-textured InP PC THz emitters is estimated to be 400 GΩ. The property of most interest is the ratio of normalized THz electric field amplitude over photocurrent, *E*_THz,0_(*V*_b_)/*I*_ph_(*V*_b_), as this will quantify the level of Joule heating during operation. It is important to note that this ratio has a direct proportionality to, and can be interpreted as, the resistance across the PC THz emitter, given that *E*_THz,0_(*V*_b_) is linearly proportional to *V*_b_. (The analyses carried out here are not subject to saturation from space-charge screening^39^ or near-field THz screening^40^ due to the low fluence levels below 50 pJ cm^−2^.) The ratio of the normalized THz electric field amplitude over photocurrent is plotted as a function of bias voltage amplitude, *V*_b_, and is plotted in Fig. 3(c) for non-, fine-, and coarse-textured InP PC THz emitters, shown respectively as black crosses, red squares, and blue circles. The measurements are the mean values for tests performed at six locations on each PC THz emitter. The error bars represent the standard error of the ratio of normalized THz electric field amplitude over the photocurrent. It is seen here that the ratio, *E*_THz,0_(*V*_b_)/*I*_ph_(*V*_b_), increases with the degree of texturing, while *E*_THz,0_(*V*_b_) remains constant, indicating that texturing decreases *I*_ph_(*V*_b_) and thus increases the resistance in the PC THz emitter. This suggests that there is a corresponding decrease in the Joule heating and ohmic loss as the degree of texturing increases for the PC THz emitter. If desired, *V*_b_ or the laser fluence can be increased for the textured PC THz emitter(s) to scale up the THz electric field amplitude. It is clear from Fig. 3(c) that the conversion between THz electric field and photocurrent increases dramatically as the PC THz emitters progress through non-, fine-, and coarse-texturing. It should be noted that non-, fine-, and coarse-textured InP PC THz emitters show an approximately linear relationship between normalized THz electric field amplitude and bias voltage amplitude right up to breakdown of the PC THz emitters. The non-textured InP PC THz emitter broke down at 100 kV cm^−1^ and the fine- and coarse-textured InP PC THz emitters broke down at approximately 110% of this value. This demonstrates a marginal improvement in electric field breakdown strength with a constant pump optical fluence. Thermal runaway is not observed at this relatively low pump optical fluence, of approximately 50 pJ cm^−2^, but it is predicted that the observed improvement will become more pronounced with higher pump optical fluences.

**Figure 4:**
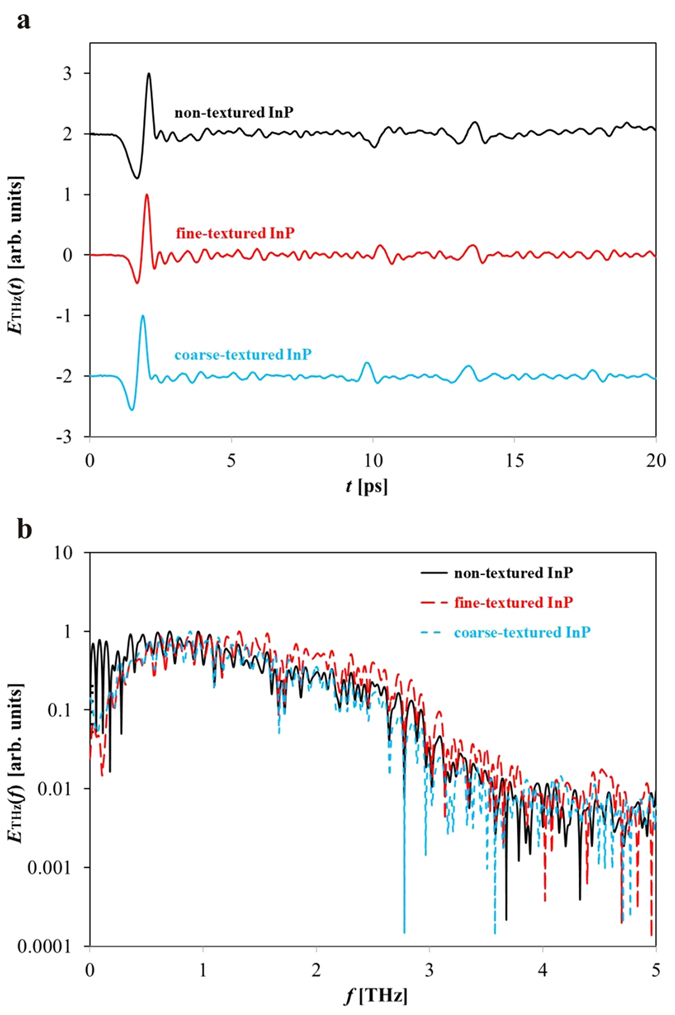
The THz electric field responses of the non-, fine-, and coarse-textured InP PC THz emitters are shown in the (**a**) time-domain and (**b**) frequency-domain. The results of (**a**) are shifted vertically for illustration purposes. Similar time-domain and frequency-domain responses are observed for the PC THz emitters. An approximate 3.5 THz spectral bandwidth is observed for each of the non, fine-, and coarse-textured InP PC THz emitters.

It is also important to test the time-domain and frequency-domain THz responses of the non-, fine-, and coarse-textured InP PC THz emitters. The THz response, *E*_THz_(*t*) (time-domain) and *E*_THz_(*f*) (frequency-domain), of the non-, fine-, and coarse-textured InP PC THz emitters are shown in Fig. 4 in the (a) time-domain as a function of time, *t*, as respective black, red, and blue solid lines, and (b) frequency-domain as a function of frequency, *f*, as respective black solid, red long-dashed, and blue short-dashed lines. It should be noted that the displayed results of Fig. 4(a) are shifted vertically for illustration purposes. Each scan is normalized with respect to its THz electric field amplitude. Similar time-domain and frequency-domain responses are observed for each PC THz emitter. The non, fine-, and coarse-textured InP PC THz emitters each have a spectral bandwidth of approximately 3.5 THz. Thus, it can be concluded that residual photocurrents can be minimized in textured PC THz emitters, for reduced Joule heating, without sacrificing the temporal and spectral characteristics of the generated THz waveforms.

It is worth noting that this work ultimately introduces a treatment (rather than a specific material) for reducing the photocurrent consumption. This treatment can be used on various semiconductor materials for varying levels of improvement in PC THz emitter performance. Here, the more standard materials for PC THz emitters are considered, and equivalent PC THz emitters made from SI-GaAs^21^,^41^, low-temperature-grown (LT-) GaAs^16^, and SI-ZnSe^42^ are compared to the non-, fine-, and coarse-textured SI-InP emitters of this work. The comparisons are made in terms of their spectral bandwidth, THz electric field amplitude, and Joule heating, while biased just below the point of dielectric breakdown. The spectral bandwidths of non-, fine-, and coarse-textured InP PC THz emitters are measured to be roughly constant at 3.5 THz. This observation is shown in the results of Fig. 4(b). The corresponding spectral bandwidths of SI-GaAs, LT-GaAs, and SI-ZnSe are found from the literature to be 3^7^, 4^43^, and 3 THz^42^, respectively. These spectral bandwidths are comparable to the values measured for the InP PC THz emitters. The emitted THz electric field amplitude is estimated according to^44^


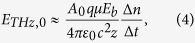


where *A*_0_ is the active area of the PC THz emitter, *q* is the elementary charge constant, *μ* is the semiconductor mobility, *E*_b_ is the (breakdown) electric field bias, *ε*_0_ is the permittivity of free space, *c* is the speed of light in free space, *z* is the propagation distance to the first parabolic mirror, Δ*n* is the change in charge-carrier density, and Δ*t* is approximated as the rise-time of the charge-carrier density. This analysis estimates the THz electric field amplitude for the non-, fine-, and coarse-textured InP and SI-GaAs PC THz emitters to be on the order of hundreds of volts per centimetre, LT-GaAs PC THz emitters to be on the order of tens of volts per centimetre, and SI-ZnSe PC THz emitters to be or the order of kilovolts per centimetre. It is worth noting that the SI-ZnSe PC THz emitter can produce a particularly high emitted THz electric field amplitude (and thus a high emitted THz power). The Joule heating in a PC THz emitter is estimated according to equation (2) which shows that Joule heating is proportional to charge-carrier lifetime and to semiconductor mobility. The level of Joule heating in the non-textured SI-InP PC THz emitter is on the order of hundreds of milliwatts while the level of Joule heating in the coarse-textured SI-InP PC THz emitter is on the order of tens of milliwatts, offering an order of magnitude improvement. (The estimated Joule heating in the fine-textured PC THz emitter is intermediate between these estimated values.) It is estimated that a corresponding SI-GaAs PC THz emitter would have Joule heating on the order of hundreds of milliwatts, a LT-GaAs PC THz emitter would have Joule heating on the order of hundreds of microwatts, and a SI-ZnSe PC THz emitter would have Joule heating as high as tens of watts (due to the very long ZnSe charge-carrier lifetime^34^). It can be concluded from this comparison that the proposed texturing treatment would not benefit all materials being used for PC THz emitters. It is the PC THz emitters being implemented with materials having long charge-carrier lifetimes, such as high-power PC THz emitters using SI-GaAs or SI-ZnSe, that will witness the greatest benefits of the proposed texturing treatment.

## Discussion and Conclusions

In this work, PC THz emitters based on InP textured materials were introduced to form reduced charge-carrier lifetimes, leading to lower Joule heating. Non-, fine-, and coarse-textured InP materials were studied through a computational analysis of SEM images and were found to have relative surface areas of 1.0 ± 0.1, 2.9 ± 0.4, and 4.3 ± 0.6, respectively. The textured surfaces exhibited significantly reduced charge-carrier lifetimes, with the non-, fine-, and coarse-textured surfaces displaying charge-carrier lifetimes of 200 ± 6 ps, 100 ± 10 ps, and 20 ± 3 ps, respectively. For similar THz field amplitudes, the fine- and coarse-textured PC THz emitters were found to consume much smaller photocurrents, for smaller Joule heating, when compared to the non-textured PC THz emitter. Such textured PC THz emitters can help prevent thermal runaway and premature device breakdown for emerging high-power and/or small-scale (e.g., lab-on-a-chip) THz technologies.

## Methods

**Figure 5:**
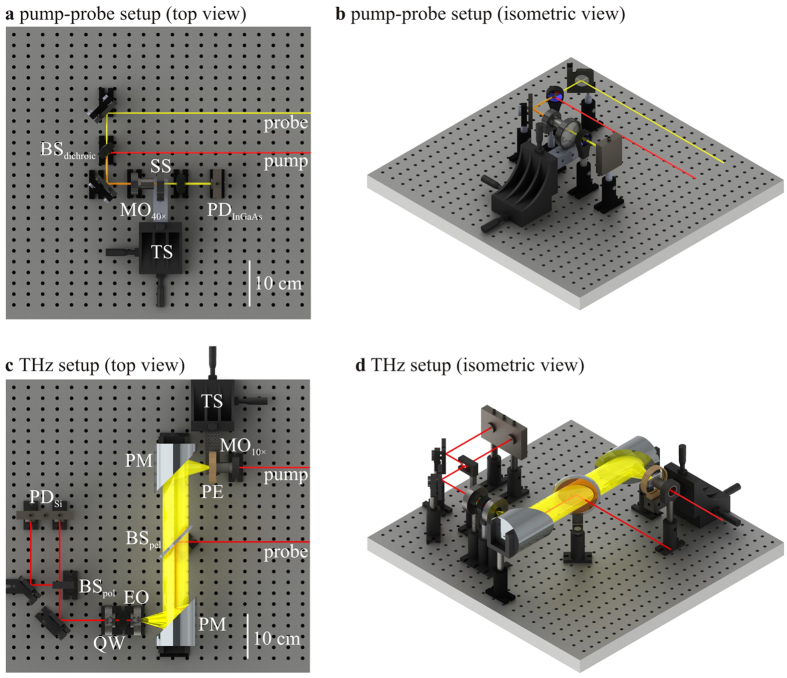
Top and isometric views of the pump-probe setup are shown in (**a**,**b**), respectively. Top and isometric views of the THz setup are shown in (**c**,**d**), respectively. The components are labeled as follows: BS_dichroic_ is a dichroic beamsplitter; MO_40×_ is a 40× microscope objective; SS is a semiconductor sample; TS is an xyz translation stage; PD_InGaAs_ is an InGaAs photodiode; MO_10×_ is a 10× microscope objective; PE is a photoconductive THz emitter; PM is a parabolic mirror; BS_pel_ is a pellicle beamsplitter; EO is an electro-optic ZnTe crystal; QW is a quarter-wave plate; BS_pol_ is a polarizing beamsplitter; and PD_Si_ is a balanced Si photodiode.

The charge-carrier lifetimes of the non-, fine-, and coarse-textured InP materials are characterized through an analysis of the ultrafast material response with a pump-probe setup. Top and isometric views of the pump-probe setup are shown in Fig. 5(a,b), respectively. Pump and probe beams, with 100 fs duration pulses^45^ and respective wavelengths of 780 and 1550 nm, are emitted from an ultrafast pulsed laser. The 780 nm wavelength is chosen for the pump beam as its 1.6 eV photon energy can photoexcite charge-carriers between the valence and conduction bands of the 1.3 eV InP bandgap with a minimal (0.3 eV) excess photon energy being dissipated as heat through phonon emission. The 1550 nm wavelength is chosen for the probe beam as its 0.8 eV photon energy is below the bandgap of InP. In the pump-probe setup, the beams are overlapped using a dichroic beamsplitter (labeled as BS_dichroic_), and focused onto the semiconductor sample (labeled as SS) using a 40× microscope objective (labeled as MO_40×_), whose position can be adjusted with an xyz translation stage (labeled as TS). Pump-induced changes to the photoconductivity of the semiconductor sample result in measurable differential probe transmission. The modulated probe powers are measured using an InGaAs photodiode (labeled as PD_InGaAs_). A second harmonic crystal and 390 nm bandpass filter can be inserted into the pump line to produce a 390 nm wavelength pump beam. The pump-probe results in this work are displayed in the form of differential transmission of the probe beam, Δ*T*(*t*)/*T*, with pump-induced changes to the transmission of the probe beam manifesting themselves through free-carrier effects. (It is assumed here that the free-carrier response dominates over the dielectric response because of the long wavelength of the 1550 nm probe beam.) Thus, there exists pump-induced free-carrier absorption (FCA), due to a change in the absorption coefficient, Δ*α*(*t*), as well as free-carrier dispersion (FCD), due to a change in the refractive index, Δ*n*(*t*). The FCA manifests itself as a change in the absorption coefficient, according to


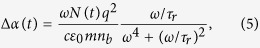


and FCD manifests itself as a change in the refractive index, according to


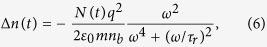


where *N*(*t*) is the photoinjected charge-carrier density, *q* is the electronic charge, *ε*_0_ is the permittivity of free-space, *m* is the effective mass, *n*_b_ is background refractive index, *ω* is the angular frequency of the probe beam, and *τ*_r_ = *μm*/*q* is the momentum relaxation time. These changes to the absorption coefficient and refractive index decrease and increase the transmission of the probe beam, respectively, which changes the differential transmission of the probe beam, according to


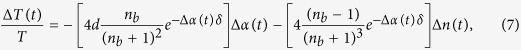


where *δ* is the penetration depth at the pump beam wavelength and *d* is the electrode gap spacing. Theoretical calculations carried out with this equation reveal that the investigated InP materials are preferentially subject to the reduction of the refractive index, and this leads to positive signals for the differential transmission of the probe beam as seen in Fig. 1. The positive polarity of these differential transmission results is also observed in experiments.

The THz response, being quantified by the radiated THz electric field and photocurrent consumption, of non-, fine-, and coarse-textured InP PC THz emitters, is characterized with a THz setup. Top and isometric views of the THz setup are shown in Fig. 5(c,d), respectively. The incident 780 nm pump beam, with 100 fs duration pulses and 50 mW power, is focused by a 10× microscope objective (labeled as MO_10×_) onto the PC THz emitter (labeled as PE), whose position can be adjusted by an xyz translation stage (labeled as TS), to form a radiated THz electric field. The PC THz emitter is fabricated from non-, fine-, and coarse-textured InP materials with approximately 100 μm spaced electrodes made with silver epoxy^41^. The THz electric field is collected by a parabolic mirror (labeled as PM) and overlapped with the 780 nm probe beam using a 2 μm thick pellicle beamsplitter (labeled as BS_pel_). The THz and probe beams are focused by a second parabolic mirror onto an electro-optic ZnTe crystal (labeled as EO) whose birefringence varies with the THz electric field according to the Pockels effect. The probe enters the quarter-wave plate (labeled as QW) and experiences THz-induced ellipticity in its polarization. The probe is then split into vertical and horizontal polarized beams by the polarizing beamsplitter (labeled as BS_pol_), and the difference of these two beams is measured using a balanced Si photodiode (labeled as PD_Si_). This provides a probe signal that is proportional to the THz electric field. While the THz electric field is being measured, a transimpedance amplifier circuit is used to measure the corresponding photocurrent of the PC THz emitter. The THz setup is designed with an especially large numerical aperture for the parabolic mirrors, being NA ≈ 0.7 as can be seen in the scaled-renderings of Fig. 5(c,d), as this allows the primary parabolic mirror to collect THz radiation that is emitted over especially broad angles. In this way, any potential dissimilarities in the emission cones of the textured InP materials will not affect the measurements.

## Additional Information

**How to cite this article**: Collier, C. M. *et al.* Photoconductive terahertz generation from textured semiconductor materials. *Sci. Rep.* **6**, 23185; doi: 10.1038/srep23185 (2016).
